# Association between tooth loss and cognitive decline: A 13-year longitudinal study of Chinese older adults

**DOI:** 10.1371/journal.pone.0171404

**Published:** 2017-02-03

**Authors:** Juan Li, Hanzhang Xu, Wei Pan, Bei Wu

**Affiliations:** 1 Nursing School of Second Military Medical University, Shanghai, China; 2 Duke University School of Nursing, Durham, United States of America; 3 Duke Global Health Institute, Durham, United States of America; 4 New York University Rory Meyers College of Nursing, New York, United States of America; University Of São Paulo, BRAZIL

## Abstract

**Objectives:**

To examine the association between the number of teeth remaining and cognitive decline among Chinese older adults over a 13-year period.

**Design:**

A large national longitudinal survey of Chinese older adults

**Setting:**

The Chinese Longitudinal Healthy Longevity Survey (CLHLS) (1998–2011).

**Participants:**

A total of 8,153 eligible participants aged 60+ interviewed in up to six waves.

**Measurements:**

Cognitive function and teeth number were measured at each interview. Cognitive function was measured by the Mini-Mental Status Examination (MMSE). Number of natural teeth was self-reported. Individuals with severe cognitive impairment were excluded. Covariates included demographic characteristics, adult socioeconomic status characteristics, childhood socioeconomic status, health conditions, and health behaviors. Linear mixed models were applied in the analysis.

**Results:**

The mean teeth number at baseline was 17.5(SD = 0.1), and the mean of baseline cognitive function was 27.3(SD = 0.0). Cognitive function declined over time (β = -0.19, *P* < .001) after controlling covariates. But, regardless of time, more teeth were associated with better cognitive function (β = 0.01, *P* < .001). The interaction of teeth number and time was significant (β = 0.01, *P* < .001), suggesting that the participants who had more teeth showed a slower pace of cognitive decline over time than those with fewer teeth after controlling for other covariates.

**Conclusion:**

This study showed that tooth loss was associated with cognitive decline among Chinese older adults. Further studies are needed to examine the linkages between cognitive decline and oral health status using clinical examination data.

## Introduction

Cognitive impairment is common among older adults. The prevalence of mild cognitive impairment (MCI) in China was 20.1% among older adults aged 60 and over in 2010 [1].

MCI constitutes an intermediate stage between normal aging and dementia, and is considered a pre-dementia syndrome [2], as the probability of MCI in adults progressing to dementia is higher than adults without MCI. The annual progression rate from MCI to dementia at 5.9% is much higher than normal cognition to dementia, of 0.6%, based on the findings from U.S studies [3], The burden of cognitive impairment in China will continue to increase in the future, as almost 290 million people are over 55[4,5].

It is important to identify risk factors of MCI in order to delay the onset and decline of cognitive impairment. Limited education, depression, chronic diseases, lack of physical activity, and poor dietary habits have all been identified as possible risk factors in previous studies[6–8]. There is increasing evidence that suggests poor oral health may be a potential risk factor for cognitive decline[6–8]. In recent years, growing number of studies conducted in developed countries have focused on the relationship between oral health and dementia or cognitive decline[6–15]. Some studies showed a negative relationship between good oral health and cognitive decline or dementia[10,13].

There is little data from longitudinal research, and studies in the field have been inconclusive in their findings. A 5-year prospective cohort study of 11,140 participants aged 55–88 with type 2 diabetes showed that those with 0 teeth or 1–21 teeth had higher risk of dementia and cognitive decline than those with 22 and more teeth [10]. However, other studies did not find the significant relationship between number of teeth and dementia or cognitive impairment [11,12,16]. An 8-year prospective cohort study of 5468 older adults showed that men and women who were edentulous or had 1–15 teeth or 16–25 teeth at baseline did not have a significantly higher risk of dementia compared to those with 26–32 natural teeth [11]. These studies used the baseline teeth number to predict the later dementia onset or cognitive decline, and the teeth number was typically coded as a categorical variable that the categorization varied by studies. Very limited studies were conducted to examine the relationship between number of teeth and the trajectory of cognitive function or decline over time. Further studies are needed to use time-specific data of teeth number and cognition and treat them as continuous variables to capture how the decrease in teeth number is related to cognitive decline. In addition, most previous studies used shorter length of the study period, while our study used 6 waves of data over a period of 13 years.

Further, no studies have examined the association between tooth loss and cognitive impairment among Chinese populations using longitudinal data. Some factors influencing oral health and cognitive function in China could be very different from those of developed countries. These factors consist of knowledge and awareness of oral health problems, oral diseases and cognitive decline, medical insurance policies, access to dental care, nutrition intake, and exposure to risk factors [17,18]. Given the rapid increase in the older adults population and related increase in the prevalence of cognitive impairment in China, it is important to use empirical evidence to examine the association between tooth loss and cognitive decline.

Our study also controlled some important covariates, such as childhood socioeconomic status (SES) in the analysis. Tooth loss is an accumulative process across life span [19]. Childhood SES has an impact on health outcomes in later life, and lower childhood SES is associated specifically with poor adult oral health [20,21]. Additionally, lower childhood SES has been found to be associated with cognitive decline in old age [22,23]. A test of tooth loss’ relationship with trajectory of cognitive function while controlling for childhood SES is particularly interesting. The purpose of this study is to explore the association between tooth number and cognitive decline among Chinese older adults over a 13-year period controlling for a number of covariates.

## Methods

### Study population

This study used Chinese Longitudinal Healthy Longevity Survey (CLHLS) data collected in the period between 1998 and 2011 with the following data collection points: 1998, 2000, 2002, 2005, 2008, and 2011. The goal of CLHLS was to better understand the determinants of healthy longevity of Chinese elderly. Participants in the CLHLS were selected from counties and cities in 22 of China’s 31 provinces. The population in these survey areas constituted approximately 85% of the total population in China. The first and second waves of CLHLS, conducted in 1998 and 2000, respectively, were limited to persons aged 80 and above. A relatively younger sample—older adults aged 60–79—were added to the CLHLS survey since 2002 and had been included in all subsequent waves. Surviving participants were re-interviewed at each follow-up wave of the CLHLS, and new participants were enrolled to make up for attrition due to loss to follow-up and death. In the CLHLS, a weight of age–sex–urban/rural residence in the sample with the distribution of the total population in the sampled 22 provinces was employed to reflect the unique sample design. Detailed information about the CLHLS sampling design and data quality were reported elsewhere [24].

Our study used a combined sample based on the five cohorts of data (1998, 2000, 2002, 2005, and 2008) to get a sufficiently large enough sample size and provide robust estimates for the 13-year study period (1998–2011) [25–27]. The first interview of each participant was at baseline, and interview of participants ranged from two to six separate waves of the data. In order to measure the association between the change in teeth number and cognitive decline, we excluded the following participants: 1) 20,643, had only one wave data due to death, loss to follow-up, or system missing; 2) 6,141, completely edentulous at initial wave; 3) 6,948, had logistic error of changes of tooth number; and 4) 262, had severe cognitive impairment with MMSE score less than ten [28]. This study included 8,153 eligible participants aged 60 and above which had at least two wave data.

The CLHLS study was approved by research ethics committees of Duke University and Peking University (IRB00001052-13074). All participants provided written informed consent. No experimental interventions were performed. An exempted Institutional Review Board (IRB) protocol was approved by Duke University (Pro00062871).

### Outcome variable

Cognitive function was measured by the Chinese version of Mini-Mental State Examination (MMSE) at each wave, which pertained to orientation, registration, attention, calculation, recall, and language [29]. MMSE score ranges from 0 to 30, with lower score indicating poorer cognition. There are three possible answers for each question: correct = 1, wrong = 0, and unable to answer. We coded responses of “unable to answer” as incorrect answers based on previous literature [23].

### Independent variable

Self-reported number of teeth was collected at each wave using the following question: “How many natural teeth do you still have?” The teeth number ranges from 0 to 32 including the third molar.

### Covariates

The main covariates were demographic characteristics, adult SES, childhood SES, health conditions and health behaviors. Demographic variables include age, gender (male = 1, female = 0), ethnicity (Han = 1, Non-Han = 0), and marital status (married = 1, others = 0). Adult SES was a continuous variable ranging from 0 to 3 with 1 point each if the respondent answered yes to the questions:(1) having 1+years education;(2) primary lifetime occupation was professional;(3) urban resident [30]. Childhood SES was a continuous variable ranging from 0 to 5 with 1 point each if the respondent answered yes to the questions: (1) obtained adequate medical services at childhood; (2) went to bed without hunger at childhood; (3) both parents were alive when the participant was age 14;(4) the participant’s father’s occupation was professional/administration; (5) place of birth was urban [22]. Health conditions included self-rated health status, self-reported chronic disease (hypertension, diabetes, heart disease, stroke or cerebrovascular disease, lung disease), and activities of daily life (ADL). Self-rated health status had two options (good = 1, poor = 0). Individual ADL was measured by the Katz index scale that consisted of six items: bathing, dressing, indoor transferring, toileting, eating, and continence [31]. The ADL disability was the number of activities that individuals needed help performing, ranging from 0 to 6. Health behaviors included current smoking (coded as 1) and current drinking (coded as 1). A time variable was also derived to indicate the time of each interview since entering into CLHLS study (time = 0,2,3,4,5,6,7,8,9,10,11,13) in relation to the CLHLS baseline and follow-up. A cohort variable was generated to indicate which cohort the participants were from.

Age, gender, ethnicity, and education, primary lifetime occupation, childhood SES were measured at baseline. All other covariates (marital status, adult SES, self-rated health, chronic diseases, ADL disabilities, current smoking and drinking levels) were measured at baseline and at each follow-up interview.

### Statistical analysis

Descriptive statistics were used to summarize baseline socio-demographic status, medical conditions, and health behaviors. A series of linear mixed models were used to estimate the association between change of teeth number and cognitive decline (time = 0,2,3,4,5,6,7,8,9,10,11,13) over time. The procedure of PROC MIXED in SAS Version 9.3 (SAS Institute Inc., Cary, NC) was used to estimate the parameters of the linear mixed models. Weights were applied to reflect the unique sampling design of the CLHLS. The missing data accounted for less than 5% of the dataset, but were not completely at random as evidenced by using Missing Completely at Random test. Therefore, a multiple imputation approach was used to reduce the potential for inferential bias and minimize the loss of subjects due to missing items [32]. All continuous variables among independent variable and covariates were centered at their grand mean values, except time and the dummy variables before entering the linear mixed models, so the estimates were more interpretable [33].

Baseline characteristics of subjects were reported as mean and standard deviation (*SD*) for continuous variables and numbers and proportion for categorical variables. All analyses were conducted using the programs of SAS 9.3 (SAS Institute, Inc., Cary, NC, USA).*P*< .01 was considered to be statistically significant because of the large sample size [34].

## Results

### Descriptive statistics

Table 1 shows the baseline socio-demographic characteristics of the study participants (N = 8153). The “unweighted” outcome in Table 1 meant the outcome from the original sample. The “weighted” outcome in Table 1 meant the outcome after using the weight to adjust the age-sex-urban/rural residence sampling strategy in order to represent the total population in the sampled 22 provinces. For the weighted outcomes, the mean age of all subjects was 74.4, and the percentage of male participants was 46.4%. About 54.8% participants were married, 50.3% participants had at least 1 year of education, and 64.6% subjects lived in rural areas. Ten percent of subjects had a professional or administrative occupation. More than half of the participants reported good health status (54.5%). The mean ADL disability was 0.1. The percentages of current smoker and drinker were 25.3% and 23.0%, respectively. The mean of baseline teeth number was 17.5(SD = 0.1), and the mean of baseline cognitive function was 27.3(*SD* = 0.0).

**Table 1:** Description of characteristics of the participants at baseline (N = 8153).

Variables	Unweighted %	Weighted%
**Age**	83.5±0.1	74.4±0.1
**Gender**		
Male	47.3	46.4
Female	52.7	53.6
**Ethnicity**		
Han	92.7	92.9
**Marital status**		
Married	38.5	54.8
**Adult Socioeconomic Status**	0.9±0.0	0.97±0.0
**Childhood Socioeconomic Status**	1.7±0.0	1.8±0.0
**Self-rated health**		
Poor	43.6	45.5
**Hypertension**	17.8	21.0
**Diabetes**	1.9	2.7
**Heart disease**	8.7	10.0
**Stroke or** cerebrovascular disease	4.2	4.8
**Lung disease**	11.4	12.1
**ADL disabilities**	0.3±0.0	0.1±0.0
**Current smoking**	21.8	25.3
**Current drinking**	23.0	23.0
**Teeth number**	13.6±0.1	17.5±0.1
**Cognitive function**	25.8±0.1	27.3±0.0

### Linear mixed model analyses

Table 2 summarizes the results of linear mixed models of multiple factors on cognitive function. Results from Model I showed that cognitive function declined with time over the follow-up period (*β* = -0.12, *P* < .001), which meant that when time increased about 1 year, the MMSE score decreased by 0.12 points. But, regardless of time, more teeth were associated with better cognitive function (*β* = 0.09, *P* < .001), which meant that for patients who had one more tooth than the other patients, their MMSE score increased by 0.09 points. Model II showed that after adding socio-demographic variables in the model, tooth number and time were still significant. Subjects who were female, older, unmarried, with lower adult SES and lower childhood SES were more likely to have poorer cognitive function. Model III showed results controlling for health status, ADL, current smoking, current drinking, and cohort effects. Participants who reported poorer self-rated health, suffered from stroke or cerebrovascular disease, or had ADL disability were more likely to have poorer cognitive function. After adding the interaction between tooth number and time, Model IV showed that cognitive function declined over time (β = -0.19, *P* < .001) after controlling for covariates, which meant that when time increased about 1 year, the MMSE score decreased by 0.19 points. Fewer teeth were associated with poorer cognitive function regardless of time (β = 0.01, *P* < .001), which meant that patients who lose one tooth, the MMSE score decreased by 0.01 points. The interaction of tooth number and time was significant (β = 0.01, *P* < .001), which meant that for patients who had one more tooth than the other patients, their MMSE score increased by 0.01 points about every year. It further suggests that the participants who had more teeth showed a slower pace of cognitive decline over time than those with fewer teeth after controlling for other covariates.

**Table 2:** The results from linear mixed models of the covariates and tooth number on cognitive function: 13-year follow-up data.

Variables	Cognitive function							
	Model I		Model II		Model III		Model IV	
	Estimate	SE	Estimate	SE	Estimate	SE	Estimate	SE
Tooth number	0.09**	0.00	0.03**	0.00	0.03**	0.00	0.01**	0.00
Time	-0.12**	0.01	-0.23**	0.01	-0.19**	0.01	-0.19**	0.01
Age			-0.15**	0.01	-0.12**	0.01	-0.12**	0.01
Male			0.91**	0.08	0.87**	0.09	0.86**	0.09
Han Ethnicity			-0.35	0.15	-0.18	0.14	-0.17	0.14
Married			0.42**	0.08	0.39**	0.07	0.40**	0.07
Adult SES			0.57**	0.04	0.60**	0.04	0.60**	0.05
Childhood SES			0.25**	0.04	0.26**	0.03	0.25**	0.04
Self-rated health					0.75**	0.06	0.75**	0.06
Hypertension					0.03	0.08	0.02	0.08
Diabetes					0.03	0.17	0.01	0.17
Heart disease					0.17	0.09	0.17	0.09
Stroke or cerebrovascular disease					-0.43*	0.13	-0.43*	0.13
Lung disease					0.06	0.09	0.06	0.09
ADL					-0.99**	0.04	-0.98**	0.04
Current smoking					-0.01	0.08	0.00	0.08
Current drinking					-0.07	0.08	-0.07	0.08
2008 cohort					0.18	0.15	0.19	0.15
2005 cohort					0.20	0.15	0.21	0.15
2002 cohort					0.17	0.13	0.18	0.13
2000 cohort					-0.31	0.13	-0.31	0.13
1998 cohort					0	0	0	0
Tooth number*time							0.01**	0.00

*p < .01,

** *p* < .001

Model I adjusted for tooth number and time. Model II adjusted for teeth number, time, age, male, Han ethnicity, marital status, adult SES and childhood SES. Model III adjusted for tooth number, time, age, male, Han ethnicity, marital status, adult SES, childhood SES, self-rated health, hypertension, heart disease, stroke or CVD, lung disease, ADL, current smoking, current drinking, and cohort effect. Model IV adjusted for tooth number, time, age, male, Han ethnicity, marital status, adult SES, childhood SES, self-rated health, hypertension, heart disease, stroke or CVD, lung disease, ADL, current smoking, current drinking, cohort effect and the interaction between tooth number and time. The 1998 cohort was the reference group for cohort effect.

## Discussion

This study is the first longitudinal study to examine the association between teeth number and cognitive decline in Chinese older adults. Teeth number and cognitive function were measured at each time point and treated as continuous variables, in order to explore the trajectory of cognitive decline and the association with tooth loss. Overall, cognitive function declined over the study period after controlling for covariates. Tooth loss was prospectively associated with cognitive decline in Chinese older adults. The higher number of tooth loss, the faster cognitive function declined. This finding added new evidence to the relationship between oral health and cognitive function.

The result of the present study is consistent with previous longitudinal studies. Case-control studies showed that history of tooth loss was a significant risk factor for poor cognitive function [6,35]. In one study, each tooth loss per decade since the baseline dental examination increased the likelihood of a low MMSE score in 597 men aged 28–70 at baseline and followed–up to 32 years in the USA [7]. Other longitudinal studies also showed the association between tooth loss and the increased risk of cognitive decline [10,13,36]. These results demonstrated that tooth loss is a risk factor for cognitive decline.

However, one longitudinal study didn’t find the association between tooth loss and cognitive decline [14]. Stewart and colleagues reported that fewer teeth was not significantly associated with cognitive decline according to quartile of teeth number at baseline (OR = 0.88, 95%CI 0.77–1.00) in a sample of 1053 Black and White men and women aged 70–79 in the USA. This inconsistency may be due to difference in race/ethnicity, age range, follow-up period, definition of cognitive decline and the statistical methods used. The follow-up period to evaluate cognitive decline in the study was short, only 2 years. The study used the quartile of teeth number at baseline and applied a logistic regression model. In contrast, our study had relatively long follow-up period (13 years), used both teeth number and cognitive function as continuous and time-varying variables, which were up to 6 waves, and applied linear mixed models. So our study may provide a more robust outcome.

There are several possible physiological mechanisms to explain the pathways between tooth loss and cognitive decline. One possibility is periodontitis, which is one of the main causes of tooth loss [37–39]. Inflammatory factors derived from the body’s response to periodontal infection may disseminate to brain through the systemic circulation and exacerbate inflammatory process and vascular pathologies [40–44]. The second possible pathway is poor nutrition due to tooth loss, including intake of insufficient recommended levels of foods, nutrients and B vitamins, which may be linked to cognitive decline [45–47]. A third possible pathway may be the decreased masticatory function as a result of tooth loss. Several clinical and animal studies had suggested that mastication was effective in sending sensory information to the brain and in maintaining learning and memory functions of the hippocampus [48–50]. Reduced masticatory function is associated with the hippocampal morphological impairments and the hippocampus-dependent spatial memory deficits, and cognitive decline especially in elderly [51].

Oral diseases are very common in China, particularly among older adults. The Third National Oral Health Epidemiological Survey in China conducted in 30 provinces had reported that 98.4% of older adults aged 65 to 74 years had cavities, and 54% of older adults thought their teeth needed to be treated [52]. But the needs for dental treatment among Chinese elderly people have been unmet. Among older adults, 98.1% of the cavities had not been treated. There are several reasons for the high percentage of oral health problems that are untreated in older adult population in China. They include a general lack of knowledge of the importance of oral health, public healthcare policy, primary care providers who are not trained in oral healthcare, and education of the general population. The percentages of older adults having a dental visit in the past 12 months were 19%. About 30% of older adults had never visited a dentist during their lifetime, and only about 7% of older adults received preventive oral services during the past one year in 2005 [53]. Only 26% of the 65-74-year-old adults brushed their teeth twice a day, and 27% of the older adults used toothpaste with fluoride [52,53]. Oral health knowledge education programs are urgently needed among older adults population. Another additional problem with good oral health care in China, is that medical insurance policies do not cover much if any dental care service. In 2003, the Urban Residents Basic Medical Insurance Scheme (URBMI) and the New Rural Cooperative Medical Scheme(NRCMS) were created by National Health and Family Planning Commission of the People’s Republic of China, but the problems of low compensation rate to healthcare providers, no reimbursement for outpatient care (including dental care) and the absence of preventative healthcare for the population insured under URBMI and NRCMS programmes, has resulted in minimal improvement of oral care [54]. About 83% of older adults must pay for the whole cost of dental care by themselves, and 26.3% of older adults are not able to afford dental treatment in 2005 [53]. There is also a shortage of trained dentists in China, 8.64 dentists per 100,000 people, which is lower than the average level in the world, 26.32 dentists per 100,000 people [55]. Establishing a preventive oral health care system, implementing community-based oral health education, oral examination and intervention, encouraging more attendance to dental schools to help bring up the dentist number, would begin to address the oral healthcare problem in China. For health care policy reform in China, it would be important to expand medical insurance coverage to include dental care.

The strengths of the present study were relatively large sample size and long follow-up period. Meanwhile, teeth number and cognitive function were measured for every wave and treated as continuous and time-varying variable, so that we could observe the trajectory of cognitive decline and the significant association between tooth loss and cognitive decline.

One limitation of the study was Mini-Mental State Examination (MMSE). MMSE is the most widely used instrument for the screening of cognitive impairment worldwide, but it is age, race, and education biased, and has a ceiling effect, which may not be sensitive for MCI [56–58]. Another limitation was self-reported teeth number. However, self-reported teeth number has been widely used as a measure of oral health in epidemiological surveys exploring the relationship between oral health and cognitive function [10,11,59,60]. Previous studies conducted in the USA demonstrated that self-reported numbers of remaining teeth was strongly correlated with clinical records(r = 0.74–1.0) [61,62]. To avoid recall bias of the participants, especially those with severe cognitive impairment, the observations’ data whose MMSE score was 0–9 indicating severe cognitive impairment in the data cleaning process, was excluded. If there was a logistic error in teeth number change, after review, the cases were excluded. Thus, the teeth number information in the study should be reliable. A total of 32 teeth (including the third molar) was used as the tooth number because extracting the third molar is not common in China. Another limitation is the reporting of general oral health in our study. There was no detailed information about periodontal disease, remaining teeth’s position and quality, denture size and type, and inflammatory biomarkers.

## Conclusion

This is the first longitudinal study reporting the association between teeth number and cognitive decline in Chinese elderly. The results from this study showed that more teeth were associated with better cognitive function, and participants who had fewer teeth tended to show a quicker pace of cognitive decline in Chinese older adults. The findings may have clinical implications on improvement of oral health and cognitive function. Further studies are needed to examine the linkages between cognitive decline and oral health status from more detailed clinical examination data.
